# The effect of food on the pharmacokinetics of oral ibrutinib in healthy participants and patients with chronic lymphocytic leukemia

**DOI:** 10.1007/s00280-015-2708-9

**Published:** 2015-02-28

**Authors:** Jan de Jong, Juthamas Sukbuntherng, Donna Skee, Joe Murphy, Susan O’Brien, John C. Byrd, Danelle James, Peter Hellemans, David J. Loury, Juhui Jiao, Vijay Chauhan, Erik Mannaert

**Affiliations:** 1 Janssen Research & Development, LLC, La Jolla, 3210 Merryfield Row, San Diego, CA 92121 USA; 2 Pharmacyclics, Inc., Sunnyvale, CA USA; 3 Janssen Research & Development, Raritan, NJ USA; 4 University of Texas, MD Anderson Cancer Center, Houston, TX USA; 5 Ohio State University, Columbus, OH USA; 6 Janssen Research & Development, Beerse, Belgium

**Keywords:** Chronic lymphocytic leukemia, Food effect, Ibrutinib, Pharmacokinetics, Tyrosine kinase inhibitor

## Abstract

**Purpose:**

To assess ibrutinib pharmacokinetics under fasted and fed conditions, impact of food-intake timing, and the safety and tolerability.

**Methods:**

Three studies were analyzed. Study 1 was a randomized, open-label, single-dose, four-way crossover study in 44 healthy participants. Study 2 was a randomized, repeat-dose crossover study in 16 patients with previously treated chronic lymphocytic leukemia (CLL). Ibrutinib dose was 420 mg in both studies. Study 3 was an open-label, sequential study to assess the effect of a standard breakfast on ibrutinib 560 mg in eight healthy participants.

**Results:**

Administration of single-dose ibrutinib under fasting conditions (study 1) resulted in approximately 60 % of exposure compared with drug intake either 30 min before, 30 min after (fed), or 2 h after a high-fat meal. Similar food effect was observed (study 3) when ibrutinib was given 30 min before meal. In CLL patients (study 2), the *C*_max_ and AUC under fasting conditions were 43 and 61 %, respectively, relative to fed conditions. When administered once-daily in uncontrolled food-intake conditions (≥30 min before or 2 h after), exposures were slightly (≈30 %) lower than in fed condition. When corrected for repeated dosing, pharmacokinetic parameters in healthy participants and patients were comparable. Ibrutinib was generally well tolerated in all settings studied.

**Conclusions:**

Ibrutinib administered in fasted condition reduces exposure to approximately 60 % as compared with dosing in proximity to food-intake, regardless of timing/type of meal. Because repeated drug intake in fasted condition is unlikely, no food restrictions may be needed to administer ibrutinib.

**Electronic supplementary material:**

The online version of this article (doi:10.1007/s00280-015-2708-9) contains supplementary material, which is available to authorized users.

## Introduction

Ibrutinib (Imbruvica^®^) was recently approved in the USA and European Union for the treatment of previously treated chronic lymphocytic leukemia (CLL) [1] and mantle cell lymphoma (MCL) [2] in patients who have had at least one prior therapy. The most common type of leukemia in the Western world is CLL and has a mean onset between 65 and 75 years of age. It is characterized by an accumulation of mature B cells in the blood, lymph nodes, and bone marrow [3]. There were an estimated 15,680 new cases of CLL among the 69,740 newly diagnosed non-Hodgkin’s lymphoma cases in the USA in 2013 [4]. Another form of non-Hodgkin’s lymphoma is MCL, which is difficult to treat and leaves patients with a poor prognosis [2, 5]. Cytotoxic chemotherapeutic agents are often used to treat B cell malignancies, and although survival may be improved, the disease is not curative [6]. Patients must be able to tolerate the side effects and toxicities associated with these agents and of particular concern, myelotoxic effects, which increases the risk of opportunistic infections. With existing frontline treatments involving multi-agent chemoimmunotherapy regimens, disease resistance to treatment is common in patients with relapse. A phase III study (RESONATE) in previously treated CLL patients demonstrated ibrutinib treatment reduced the risk of progression or death by 78 % compared to the CD20-directed monoclonal antibody, ofatumumab [7]. The safety profile was acceptable with no increase in the risk of grade 3/4 adverse events (AEs) with ibrutinib. Furthermore, despite advances, most patients with CLL and MCL die of their disease [2, 8, 9]. Therefore, novel therapies are needed for improved medical treatment for these malignant diseases.

Many factors contribute to the viability of B cells and their development, proliferation, and survival, including B-cell receptor (BCR) activation and signaling [10]. The BCR signaling is a key factor in the survival of B cell malignancies [11–13]. Although the exact mechanism by which BCR regulates B cell activity is unknown, BCR activation is mediated via a cascade of enzymatic activity, among which is an essential kinase enzyme, Bruton’s tyrosine kinase (BTK) [14, 15]. Bruton’s tyrosine kinase is expressed in all hematopoietic cells, with the exception of T lymphocytes and natural killer cells [16]. Dysfunctional BTK, due to mutations, leads to an inherited disease, X-linked agammaglobulinemia, in which patients lack peripheral mature B cells, have increased serum immunoglobulin, and are more susceptible to infection [16, 17]. Therefore, it is reasonable to target BTK to inhibit survival and proliferation of malignant B cells.

Preclinical studies have demonstrated that ibrutinib inhibits several processes, including survival, proliferation, adhesion, and tumor cell migration [18–20]. Inhibition of BTK is a novel mechanism to treat non-Hodgkin’s lymphoma, and ibrutinib does this by forming an irreversible covalent bond with BTK at the Cys 481 site. In early studies, ibrutinib as a single agent demonstrated high response rates and durable efficacy in previously treated B cell malignancies [1, 2, 21].

As ibrutinib is orally administered, it is important to determine the relative bioavailability in relation to meal consumption. Typical for first-in-human studies in a patient setting, and because the absence or presence of a food effect had not been confirmed in preclinical studies, it was deemed advisable to avoid both fully fasted and fed conditions in studies supporting the early clinical development of ibrutinib [22].

The primary objective of the current analysis was to assess the effect of food on the pharmacokinetics (PK) of ibrutinib at 420 mg, the therapeutic dose for CLL, as well as to investigate the effects of food timing and type of meal. Secondary objectives included determining the PK of ibrutinib metabolite PCI-45227 and assessing the safety and tolerability of ibrutinib administered in single doses up to 560 mg.

## Materials and methods

### Study design and treatment

Two studies were designed to evaluate the effect of a high-fat meal on ibrutinib exposure. A third, exploratory, study tested the effect of a standard breakfast. Study 1 (NCT01820936) was a randomized, open-label, single-center, single-dose, four-way crossover study in 44 healthy participants. Study 2 (NCT01105247) was a two-center, randomized (fasted vs. fed), repeat-dose crossover substudy in 16 patients with previously treated CLL who were able to roll over to a long-term extension study after 6 months of treatment (NCT01109069). Study 3 (NCT01866033) was an open-label, single-center, single-dose, sequential study in eight healthy participants.

Healthy participants received a single, oral ibrutinib 420 mg (study 1) or 560 mg dose (study 3) on day 1 of each treatment period; patients with previously treated CLL (study 2) received ibrutinib 420 mg daily. For all treatment arms, participants were confined in the study center during PK blood sample collection (72 h: studies 1 and 3; 24 h: study 2). In studies 1 and 3, there was a washout period of 7 days between dosing in subsequent periods. In study 2, as in all other ibrutinib studies in patients, investigators instructed patients to take the drug, per protocol, at least 30 min before or 2 h after a meal on nonsampling days. Patients were allowed to take their concomitant medications on all days. In addition to the formal (fasted/fed) PK evaluations in study 2 under repeat-dosing conditions, an uncontrolled food timing evaluation was performed on day 1 under single-dose condition. As the day 1 PK evaluation was uncontrolled, no formal statistical comparisons were performed between single-dose and repeat-dose conditions.

**Fig. 1:**
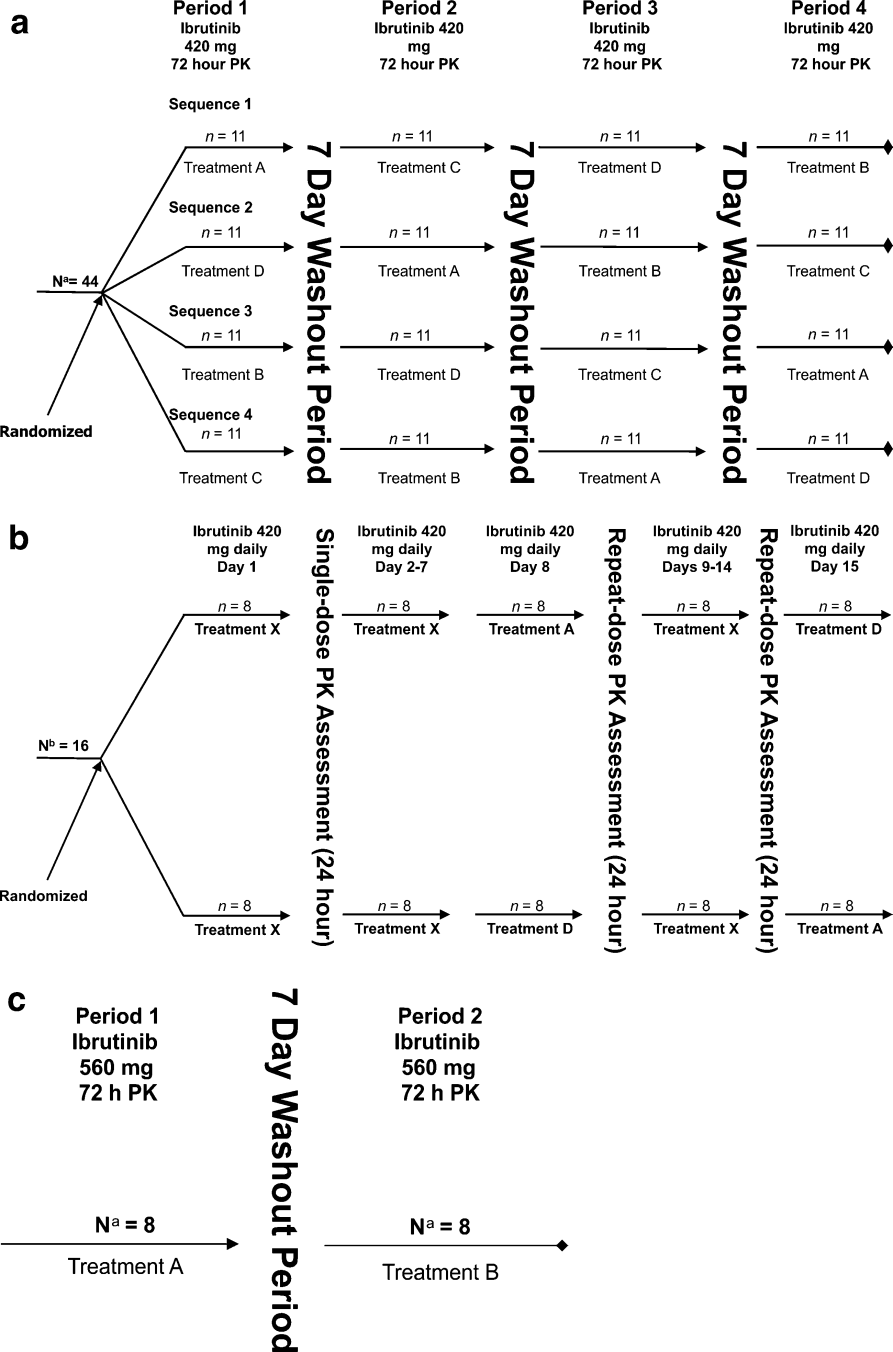
Treatment sequence. **a** Study 1: healthy participants receiving single-dose oral ibrutinib, **b** study 2: patients with previously treated chronic lymphocytic leukemia receiving repeat-dose oral ibrutinib, **c** study 3: healthy participants receiving single-dose oral ibrutinib. *PK* pharmacokinetics. Treatment A = Ibrutinib orally administered after fasting for ≥10 and 4 h before the next food-intake. Treatment B = Ibrutinib orally administered after fasting for ≥10 h and 30 min before a meal. Treatment C = Ibrutinib orally administered 2 h after a meal. Treatment D = Ibrutinib orally administered 30 min after completing a meal. Treatment X = Ibrutinib orally administered at least 30 min before or at least 2 h after a meal. ^a^Healthy participants receiving single-dose study drug. ^b^Patients with previously treated chronic lymphocytic leukemia receiving repeat-dose study drug

All treatment regimens started with an overnight fast (≥10 h), followed by a controlled combination of ibrutinib and food-intake. All studies explored a controlled fasting state (Treatment A). Study 1 explored dosing/food-intake regimens, including ibrutinib 30 min before a meal (treatment B); 30 min after completing a meal (fed, treatment D); and 2 h after a meal (treatment C). Study 2 utilized fasting state (treatment A) and fed state (treatment D) and also evaluated PK on day 1 where patients were allowed to take ibrutinib at uncontrolled food-intake conditions (at least 30 min before or 2 h after meal; treatment X). Study 3 utilized treatment A and treatment B (30 min before meal). Treatment descriptions are shown in Supplementary Table 1 and treatment sequences in Fig. 1a–c.

In studies 1 and 2, the test meal was composed of 800–1000 calories, approximately 50 % of which was derived from fat and was to be consumed in 30 min. In study 3, during treatment B, ibrutinib was given 30 min after a sugary drink; participants then consumed a standard fat meal within 20 min. In study 1, water was allowed 2 h after ibrutinib administration. In studies 2 and 3, water was allowed up to 1 h before and after ibrutinib administration, and 1 h before and 2 h after administration, respectively. In all studies, healthy participants and patients took ibrutinib with 240 mL of water and a meal was provided approximately 4 h after ibrutinib administration.

### Eligibility

In studies 1 and 3, participants included healthy men and women 18–55 years of age who had a body mass index (BMI) between 18 and 30 kg/m^2^ (inclusive) and weight ≥50 kg.

Participants were excluded for prior or current illness that could interfere with the interpretation of the results; any clinically significant abnormal findings in laboratory values for hematology or chemistry, urinalysis, physical examination, or vital signs; use of prescription or nonprescription medications including nonsteroidal anti-inflammatory drugs ≤7 days prior to screening; use of herbal supplements 30 days prior to the study or vitamins ≤14 days prior to administration of the first dose of the study drug (acetaminophen or hormonal replacement therapy were permitted); history of drug or alcohol abuse; known allergy to the study drug or excipients of the formulation; recent blood donation or loss of blood volume of ≥500 mL; positive test for hepatitis B or C or human immunodeficiency virus infection; use of nicotine in the past 2 months; or planned surgery. Eligibility criteria for study 2 are previously published [1]. The key inclusion criteria included a diagnosis of relapsed or refractory CLL or small lymphocytic lymphoma, as defined according to the International Workshop on CLL and World Health Organization classifications and a need for treatment.

### Pharmacokinetic assessments

Plasma and urine concentrations of ibrutinib and its metabolite PCI-45227 were measured using liquid chromatography with tandem mass spectrometry. The ibrutinib PK parameters across all studies included maximum observed plasma concentration (*C*_max_), time to *C*_max_ (*t*_max_), area under the plasma concentration–time curve (AUC) from time 0 to the time of last quantifiable concentration (AUC_last_), AUC from 0 to 24 h (AUC_0–24h_), and elimination half-life (*t*_1/2_).

### Safety assessments

In studies 1 and 3, clinical laboratory tests including hematology, platelet function assay (PFA-100), serum chemistry, urinalysis, electrocardiogram (ECG), physical examination, and vital signs were assessed. A follow-up visit was completed at 10 ± 2 days after the last dose to evaluate lymphocyte count and any additional AEs. Study 2 safety assessments have previously been described [1].

### Sample collection

In studies 1 and 3, blood samples (2 mL) for PK evaluation of ibrutinib and its metabolite PCI-45227 were collected at predose and 0.5, 1, 1.5, 2, 3, 4, 6, 8, 12, 16, 24, 36, 48, and 72 h postdose during each treatment period. In study 2, blood samples (2 mL) were collected at predose and 0.5, 1, 2, 4, 6, and 24 h after ibrutinib administration, and at the same time points for the formal food effect comparison at steady state on days 8 and 15.

### Statistical analyses

For PK parameters, linear mixed-effect models were applied in log-transformed PK parameters (*C*_max_, AUC_last_, and AUC_0–24h_) with treatment, period, and treatment sequence as fixed effects and participants as random effect. Geometric mean ratios (GMRs) between tests and references of *C*_max_ and AUC_last_ and the corresponding 90 % confidence intervals (CIs) were constructed following back-transformation. Descriptive statistics were used to describe PK parameters. Non-compartmental analysis with WinNonlin Professional software version 5.2.1 (Pharsight Corp, Mountain View, CA, USA) was used to calculate PK parameters for ibrutinib and metabolite PCI-45227. Concentrations below the lower level of quantifiable concentration were treated as zero in the summary statistics.

In studies 1 and 3, descriptive statistics were used for age, BMI, weight, and height in participants who received ≥1 dose of study drug. All participants who received ≥1 dose of study drug were included in the safety analyses. All AEs were recorded and followed throughout the study and follow-up.

In study 1, a sample size of 44 participants was planned based on statistical estimation to enable the study to provide an estimate on the magnitude of food effect with precision close to the limit of 80–125 %.

## Results

### Participant’s disposition and baseline characteristics

Of the 44 participants (38 men) enrolled in study 1 (Table 1), one participant prematurely discontinued (withdrew consent) after period 1 and was not included in the PK analysis. The median (range) age was 39 (24–55) years. Baseline demographics were comparable across treatment groups. In study 2, all 16 patients (14 men) with previously treated CLL completed the study. Patients were 51–80 (median 62) years of age. Additional baseline characteristics for patients in study 2 have previously been reported [1]. In study 3, all eight participants (3 men) completed the study (Table 1).

**Table 1:** Baseline characteristics of participants from studies 1 and 3

Demographic/characteristics	Study 1 (*n* ^a^ = 44)	Study 3 (*n* ^a^ = 8)
Age (years)		
Mean (SD)	38.5 (9.7)	46.4 (8.1)
Median (range)	39.0 (24–55)	48.5 (34–55)
Men, *n* (%)	38 (86.4)	3 (37.5)
Race, *n* (%)		
White	12 (27.3)	8 (100)
Asian	1 (2.3)	0
Black or African American	29 (65.9)	0
Multiple	2 (4.5)	0
Ethnicity, *n* (%)		
Hispanic or Latino	9 (20.5)	0
Not Hispanic or Latino	35 (79.5)	8 (100)
Weight (kg)		
Mean (SD)	79.3 (10.7)	70.0 (13.2)
Median (range)	80.1 (54.7–96.9)	69.7 (50.7–90.6)
Height (cm)		
Mean (SD)	174 (9.0)	172 (11.3)
Median (range)	174 (154–196)	170 (160–189)
BMI (kg/m^2^)		
Mean (SD)	26.2 (2.5)	23.5 (2.7)
Median (range)	26.9 (19.7–29.4)	23.0 (19.6–27.4)

*BMI* body mass index, *SD* standard deviation

^a^Healthy participants receiving single-dose study drug

### Intra-study pharmacokinetic results

**Fig. 2:**
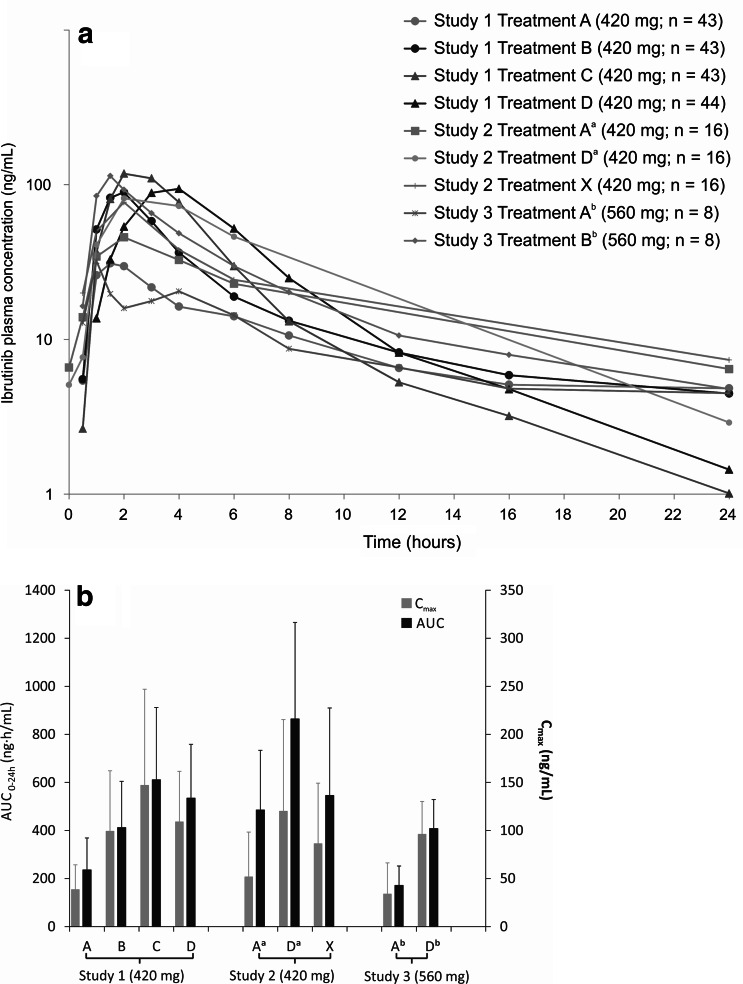
**a** Log-linear time versus concentration curve in plasma following 420 and 560 mg oral ibrutinib administration with various meal and meal-time adjustments in healthy participants and patients with chronic lymphocytic leukemia. **b** Cross-study comparisons of *C*_max_ and AUC in fed and fasting conditions. *AUC* area under the plasma concentration–time curve, *C*_*max*_ maximum observed plasma concentration. Treatment A = Ibrutinib orally administered after fasting for ≥10 and 4 h before the next food-intake. Treatment B = Ibrutinib orally administered after fasting for ≥10 h and 30 min before starting a meal. Treatment C = Ibrutinib orally administered 2 h after a meal. Treatment D = Ibrutinib orally administered 30 min after completing a meal. Treatment X = Ibrutinib orally administered at least 30 min before or at least 2 h after a meal. ^a^Study 2 treatments A and D dose obtained at steady state, all others after single-dose. ^b^Dose normalized to a 420 mg dose

Ibrutinib concentration time curves and cross-study comparisons of *C*_max_ and AUC in fed and fasting conditions are shown in Fig. 2a, b, and Table 2 lists PK parameters across the studies. In study 1, ibrutinib *C*_max_ and AUC GMR in the fed condition (treatment D, 30 min after a meal) were 3.15 and 1.86, respectively, compared with the fasting state (treatment A). When timing of ibrutinib changed from the fed condition to 2 h after a meal (treatment C), *C*_max_ and AUC GMR were 3.85 and 1.78, respectively. In studies 1 and 2, under fed conditions (treatment D), the ibrutinib AUC_last_ GMRs were similar (1.86 vs. 1.65, respectively), while the ibrutinib *C*_max_ GMR was slightly higher in study 1 than in study 2 (3.15 vs. 2.24). In study 1, exposure measured as AUC_last_ was lower in the fasted condition (treatment A), relative to exposure with dosing within 30 min to 2 h of food-intake. The AUC_last_ in the fasted state (treatment A) was 63.2 % compared with dosing 30 min before a meal (treatment B), 55.5 % versus dosing 2 h after a meal (treatment C), and 56.2 % versus dosing 30 min after a meal (treatment D). The *C*_max_ and AUC values for uncontrolled food timing on day 1 (≥30 min before or 2 h after; treatment X) in study 2 were intermediate to those of fed (treatment D) and fasted conditions (treatment A). The *C*_max_ and AUC in fasting conditions were 43 and 61 %, respectively, relative to fed conditions. In study 3, *C*_max_ GMR was 3.52 and AUC_last_ GMR was 2.23, for dosing in the pre-fed condition (treatment B) as compared to the fasted condition.

**Table 2:** Ibrutinib pharmacokinetic parameters in healthy participants and patients with chronic lymphocytic leukemia following oral ibrutinib administration in all treatments

	*N*	*C*_max_ (ng/mL)	*C*_max_ GMR (90 % CI)	*t*_max_ (h), median (range)	AUC_0–24h_ (h·ng/mL)	AUC_last_ (h·ng/mL)	AUC_last_ GMR (90 % CI)	*t*_1/2, term_ (h)
Study 1^a^								
Treatment A	43	38.5 (25.8)	NA	1.5 (1.0–8.0)	236 (133)	289 (163)	NA	9.7 (3.2)^c^
Treatment B	43	99.2 (62.9)	2.63 (226.6, 304.5)	1.5 (1.0–4.0)	412 (193)	457 (213)	1.62 (147.6, 178.3)	9.0 (3.3)^d^
Treatment C	43	147 (100)	3.85 (332.2, 446.5)	3.0 (1.0–6.0)	611 (301)^e^	521 (301)	1.78 (161.7, 195.4)	5.2 (1.9)^f^
Treatment D	44 ^g^	109 (52.6)	3.15 (271.7, 364.9)	4.0 (2.0–6.0)	535 (224)^h^	514 (237)^i^	1.86 (169.1, 204.2)	4.8 (1.4)^i^
Study 2^b^								
Treatment A	15	51.7 (46.7)	NA	1.9 (0.9–4.1)	485 (249)^j^	455 (265)	NA	11 (9.6)^k^
Treatment X	16	86.3 (63.0)	NA	2.0 (1.0–4.0)	546 (364)	546 (364)	NA	5.6 (1.2)^l^
Treatment D	16	120 (95.4)	2.24 (161.6, 309.4)^m^	3.9 (1.1–6.0)	864 (402)^n^	750 (436)	1.65 (123.4, 219.4)^m^	4.5 (0.8)^o^
Study 3^a^								
Treatment A	8	45.2 (43.3)	NA	3.8 (0.5–5.0)	229 (107)	289 (117)	NA	13 (4.9)^o^
Treatment B	8	128 (45.6)	3.52 (212.7, 581.6)	1.8 (1.5–5.0)	544 (161)^p^	606 (160)	2.23 (167.0, 297.3)	9.5 (4.1)^p^

Data presented as mean (SD), unless otherwise specified

*AUC*_*last*_ area under the plasma concentration–time curve from time 0 to the time of last quantifiable concentration, *AUC*_*0*–*24h*_ area under the plasma concentration–time curve from time 0–24 h, *CI* confidence interval, *CL* total clearance, *C*_*max*_ maximum observed plasma concentration, *GMR* geometric mean ratio, *NA* not applicable, *SD* standard deviation, *t*_*max*_ time to reach *C*_max_, *t*_*1/2,term*_ terminal elimination half-life

Treatment A = Ibrutinib orally administered after fasting for ≥10 and 4 h before the next food-intake (=reference treatment)

Treatment B = Ibrutinib orally administered after fasting for ≥10 h and 30 min before a meal

Treatment C = Ibrutinib orally administered 2 h after a meal

Treatment D = Ibrutinib orally administered 30 min after completing a meal

Treatment X = Ibrutinib orally administered at least 30 min before or at least 2 h after a meal

^a^Healthy participants receiving single-dose study drug

^b^Patients with previously treated chronic lymphocytic leukemia receiving repeat-dose study drug

^c^ *n* = 27

^d^ *n* = 36

^e^ *n* = 30

^f^ *n* = 39

^g^One participant not included in descriptive statistics

^h^ *n* = 38

^i^ *n* = 42

^j^ *n* = 14

^k^ *n* = 9

^l^ *n* = 6

^m^ *n* = 15

^n^ *n* = 13

^o^ *n* = 4

^p^ *n* = 7

**Fig. 3:**
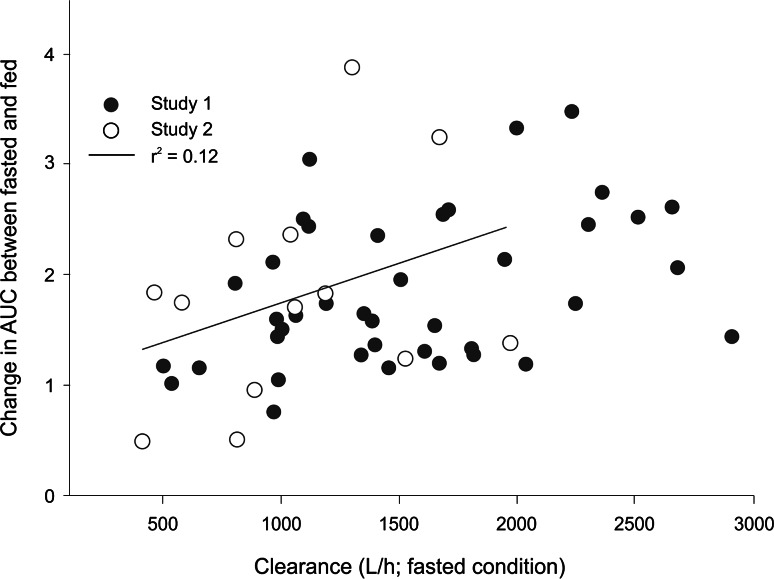
Correlation between clearance rate of ibrutinib and food effect on AUC

In studies 1 and 2, under fasted conditions, ibrutinib was rapidly absorbed after oral administration with a median *t*_max_ of 2 h while the median *t*_max_ under fed conditions was 4 h. In line with the variability in exposures, a wide range of apparent clearance values was observed for both study 1 and 2 (Fig. 3).

In study 1, compared with the fasted condition, PCI-45227 concentrations were higher when ibrutinib was administered with a meal, and *C*_max_ was highest when ibrutinib was given 30 min after a meal. Compared with the fasted condition, PCI-45227 mean *C*_max_ was 1.7–2.9 times higher, and AUC was 1.4–2.3 times higher when dosed with a meal. Half-life remained similar across all treatments. A trend in metabolite-to-parent ratios could not be identified. Similarly in study 3, PCI-45227 exposure increased (approximately 1.6-fold) when ibrutinib was administered with food compared with the fasting condition. In studies 1 and 2, the PCI-45227 mean *t*_max_ was achieved in 4 h under fed conditions (treatment D) compared with 2 h in the fasted condition (treatment A).

### Safety

Across all studies, there were no serious treatment-emergent adverse events (TEAEs) related to ibrutinib. During the food effect evaluation, no patients in study 2 discontinued treatment or required dose reduction due to a TEAE. There were no deaths during food effect evaluation.

In study 1 (single-dose, 420 mg ibrutinib), 13 (29.5 %) participants reported ≥1 TEAE (Table 3). All TEAEs were of grade 1 severity except for one participant who experienced a grade 2 TEAE of viral syndrome. The most common TEAEs (*n* = ≥3) were diarrhea and headache [3/44 (6.8 %), each]. Overall, there were no clinically significant observations in ECG, vital signs, or laboratory values (data not shown).

**Table 3:** Treatment-emergent adverse events in ≥15 % of population

Adverse event	Study 1 (*n* ^a^ = 44)	Study 2 (*n* ^b^ = 16)	Study 3 (*n* ^a^ = 8)
Number of healthy participants/patients with TEAEs	13 (29.5)	16 (100.0)	3 (37.5)
Abdominal pain	1 (2.3)	0	3 (37.5)
Diarrhea	3 (6.8)	11 (68.8)	3 (37.5)
Vomiting	0	4 (25.0)	1 (12.5)
Dizziness	1 (2.3)	0	2 (25.0)
Headache	3 (6.8)	4 (25.0)	1 (12.5)
Hyperventilation	0	0	1 (12.5)
Arthralgia	0	5 (31.3)	0
Contusion	0	4 (25.0)	0
Epistaxis	0	4 (25.0)	0
Fatigue	0	4 (25.0)	0
Pneumonia	0	4 (25.0)	0
Upper respiratory tract infection	0	4 (25.0)	0
Chills	0	3 (18.8)	0
Increased tendency to bruise	0	3 (18.8)	0
Muscle spasms	0	3 (18.8)	0
Pain in extremity	0	3 (18.8)	0

Data shown as *n* (%)

*TEAE* treatment-emergent adverse event

^a^Healthy participants receiving single-dose study drug

^b^Patients with previously treated chronic lymphocytic leukemia receiving repeat-dose study drug

In study 2 (repeat-dose, 420 mg ibrutinib), during the food effect evaluation, all 16 patients experienced at least 1 TEAE. A total of 18.8 % of patients had experienced grade ≥3 that was considered related. The most frequent serious adverse event (SAE) in this heavily pretreated population was pneumonia. In study 3 (single-dose, 560 mg ibrutinib), two participants experienced TEAEs, including lightheadedness (*n* = 2), abdominal cramps (*n* = 2), and diarrhea (*n* = 1). These events were grade 1 in severity except for grade 2 abdominal cramps in one participant. Overall, there were no clinically significant observations in ECG, vital signs, or laboratory values (data not shown).

## Discussion

These three studies sought to understand the impact of food on the relative bioavailability of oral ibrutinib (420 and 560 mg) by evaluating the effect of three meal times and a fasting state. Food can change the bioavailability of a drug and thus can have clinical consequences. Specifically, food effects on bioavailability are greatest when a drug is administered within a short time period in relation to a meal. Some of the ways food affects bioavailability are through delayed gastric emptying, changes in gastrointestinal pH, or improved solubility. Another effect of food-intake is increased splanchnic blood flow. As patient instructions for clinical studies suggest ibrutinib to be taken at least 30 min before or 2 h after a meal, these scenarios were used to determine the relationship between ibrutinib and food. To properly evaluate this relationship, single-dose ibrutinib was analyzed under various timing sequences in relation to food. The results of these analyses were then compared with the PK of repeat-dose ibrutinib administration in clinical patients with refractory CLL.

In study 1, race was primarily represented by black or African American populations, while in studies 2 and 3, the population was primarily white. The median age and weight of patients with CLL in study 2 were greater than that of healthy participants. However, population PK analysis across three patient studies did not suggest an effect of these covariates on ibrutinib clearance [23]. An exploratory comparison between the white and black participants in study 1 did not suggest an effect of race (data not shown). Therefore, results could be comparable between studies.

Administration of ibrutinib in a fasted condition resulted in approximately 60 % of plasma exposure (AUC_last_) as compared with administration either 30 min before or 2 h after a meal. Because ibrutinib is a high-clearance drug, known to be mediated by CYP3A and limited by blood flow, the observed food effect is most likely caused by an increase in intestinal blood flow rather than increased absorption. The higher blood flow increases the passage from the intestine into the portal circulation, thus decreasing the first-pass effect caused by intestinal CYP3A. The higher the intestinal CYP3A activity, the more pronounced the blood flow-mediated effect of food on first-pass metabolism. The result of this effect can be observed in the potential positive trend when comparing the food effect in individual participants with baseline ibrutinib clearance (Fig. 3).

Elimination half-life appeared shorter (approximately 5 h) when ibrutinib was administered after the meal compared with dosing under fully fasted or 30 min before meal conditions (9–13 h). It can be speculated that flip-flop kinetics occur here: when the dose of drug follows food-intake by either 30 min or 2 h after drug intake, delayed absorption may be the result. Under fasted conditions, ibrutinib concentrations were much more variable. A secondary peak was observed during the distribution phase, which was not observed when given with food. The second peak coincides with lunch time, and this meal may trigger bile secretion from the gall bladder, accelerating solubility of the drug already in the gastrointestinal lumen. The exposure to metabolite PCI-45227 was also higher in the fed state than fasted state for both *C*_max_ and AUC. Half-life, however, was similar under all conditions. There was no definitive trend for metabolite–parent ratio.

Safety results from the food effect evaluation in study 2 (with CLL patients) were comparable to those of the larger study population [1]. Across studies 1 and 3 in healthy participants, the safety profile of ibrutinib was similar with all treatments. In addition, there were no clinically important treatment-emergent changes or adverse trends in hematology, clinical chemistry, urinalysis, ECG, or vital signs. Overall, ibrutinib had an acceptable safety profile.

As ibrutinib is a novel therapy in CLL and MCL treatment, with a unique mechanism of action, it is important to continue investigating this therapy.

In conclusion, single oral doses of ibrutinib 420 and 560 mg and repeat doses of 420 mg had acceptable safety profiles in healthy participants and patients with previously treated CLL. After administration of ibrutinib in fully fasted conditions, plasma exposure (AUC) is about 60 % of what is observed when dosing was in proximity to a meal (from 30 min before to 2 h after food-intake). Considering the favorable safety profile and the unlikeliness of repeated intake in fasted conditions, no food restrictions are needed to administer ibrutinib. Thus, ibrutinib is registered in the USA and European Union for administration regardless of food intake.

## Electronic supplementary material

Below is the link to the electronic supplementary material.

Supplementary material 1 (DOCX 14 kb)
