# Supplementary material for: The effect of food on the pharmacokinetics of oral ibrutinib in healthy participants and patients with chronic lymphocytic leukemia
Source: Cancer Chemother Pharmacol. 2015 Feb 28;75(5):907–16. doi: 10.1007/s00280-015-2708-9 (PMC4419161; doi:10.1007/s00280-015-2708-9)
Supplement: Supplementary file 1 — Supplementary material 1 (DOCX 14 kb) [file 280_2015_2708_MOESM1_ESM.docx]

**Supplementary Data:**

**Table S1.** Treatment description

| Treatment A | Ibrutinib orally administered after fasting for ≥10 hours and 4 hours before the next food intake |
| --- | --- |
| Treatment B | Ibrutinib orally administered after fasting for ≥10 hours and 30 minutes before a meal |
| Treatment C | Ibrutinib orally administered 2 hours after a meal |
| Treatment D | Ibrutinib orally administered 30 minutes after completing a meal |
| Treatment X | Ibrutinib orally administered at least 30 minutes before or 2 hours after a meal |

**Table S2:** PCI-45227 pharmacokinetic parameters (study 1)

|  | **Treatment A (n = 43)** | **Treatment B (n = 43)** | **Treatment C (n = 43)** | **Treatment D (n = 43)** |
| --- | --- | --- | --- | --- |
| *C_max_* (ng/mL) | 54.3 (20.8) | 92.0 (33.7) | 158 (34.9) | 126 (27.4) |
| *C_max_* M/P | 1.60 (0.6) | 1.03 (0.4) | 1.35 (0.9) | 1.22 (0.5) |
| *t_max_* (h), median (range) | 2.00  (1.00 - 8.00) | 2.00 (1.00 - 6.00) | 3.00 (1.50 - 6.00) | 4.00 (1.50 - 8.00) |
| *AUC_0-24h_* (h.ng/mL) | 538 (197) | 809 (236) | 1255 (249) | 1136 (207) |
| *AUC_0-24h_* M/P | 2.42 (0.9) | 2.04 (0.6) | 2.29 (0.8) | 3.27 (0.8) |
| *AUC_last_* (h.ng/mL) | 692 (245) | 997 (274) | 1442 (320) | 1328 (267) |
| *AUC_last_* M/P | 2.55 (0.9) | 2.28 (0.8) | 3.21 (1.6) | 2.69 (0.9) |
| Data shown as mean (standard deviation), unless otherwise specified. *AUC_last_* area under the plasma concentration-time curve from time 0 to the time of last quantifiable concentration, *AUC_0-24h_* area under the plasma concentration-time curve from time 0 to 24 hours, *C_max_* maximum observed plasma concentration, *M/P* metabolite/parent ratio, *t_max_* time to reach *C_max_*  ^a^ n = 42, ^c^ n = 41.  Treatment A = Ibrutinib orally administered after fasting for ≥10 hours and 4 hours before the next food intake.  Treatment B = Ibrutinib orally administered after fasting for ≥10 hours and 30 minutes before starting a meal.  Treatment C = Ibrutinib orally administered 2 hours after a meal.  Treatment D = Ibrutinib orally administered 30 minutes after completing a meal. | | | | |
